# Correspondence

**Published:** 1914-02

**Authors:** Robt. J. Talbot

**Affiliations:** Niagara Falls, N. Y.


### CORRESPONDENCE.

*This department is intended for the presentation of news and views not coming within the scope of other departments, and particularly to afford opportunity for discussion and criticism of views elsewhere expressed.*

Niagara Falls, N. Y., Dec. 30, 1913.

Editor, Buffalo Medical Journal,
Buffalo, N. Y.

Dear Sir—Regarding the attack on the Dowd Phosphatometer, A· M. A., December 20th, 1913, it is evident on the face of it that those of us who have used the Phosphatometer and observed the great variations in the amount of phosphates present in different conditions of illness and the almost immediate results obtained following treatment as directed by its readings that instead of "the thinking men believing it an unscientific absurdity," as they term it, they are the ones that should be deprived at once of their license to think.

I shall agree with them that it may as yet not be on a highly scientific basis, for I think there is still much to learn regarding the Phosphatic Index, but as one Doctor expressed himself some time ago in a medical article, speaking of things that as yet may be considered empirical, "We cannot abstain from or discard a useful method until the precise explanation is revealed to us and we cannot forget that empiricism has often anticipated science in Medicine." This may be made more clear if I say, that for the past 400 years all persons infected with syphilis should have been allowed to suffer or die because we did not thoroughly understand the action of mercury on the disease.

Regarding a quantitative analysis, how about sugar, albumin, etc. Must we wait for a 24-hour specimen of urine from each patient before prescribing? A diabetic might pass into coma from which he would never return while waiting for the 24-hour specimen. Possibly only a part of the phosphates may pass out through the urine, the same may be said of albumin, sugar, etc. Must we stop examining for these substances? The rise and fall of the phosphates under appropriate medication, as suggested by the phosphatometer, will be evidenced to any one at once upon using this instrument and instead of it being "an unscientific humbug" they are the ones that should be so classed. I can only substantiate Dowd's opinion regarding the Co. Phos. Tonic, it does just what he says it does.

Niagara Falls, N. Y. ROBT. J. TALBOT.

